# An emergency brake for protein synthesis

**DOI:** 10.7554/eLife.27085

**Published:** 2017-04-25

**Authors:** Vladislava Hronová, Leoš Shivaya Valášek

**Affiliations:** Laboratory of Regulation of Gene Expression, Institute of Microbiology ASCR, Prague, Czech Republic; Laboratory of Regulation of Gene Expression, Institute of Microbiology ASCR, Prague, Czech Republicvalasekl@biomed.cas.cz

**Keywords:** translational control, eIF2, eIF2B, protein interactions, ISR, *S. cerevisiae*

## Abstract

The integrated stress response is able to rapidly shut down the synthesis of proteins in eukaryotic cells.

**Related research article** Jennings MD, Kershaw CJ, Adomavicius T, Pavitt GD. 2017. Fail-safe control of translation initiation by dissociation of eIF2α phosphorylated ternary complexes. *eLife* **6**:e24542. doi: 10.7554/eLife.24542

When driving a car it is usually best to brake gently when you want to stop. Occasionally, however, it is necessary to 'stand on the brakes' and perform an emergency stop. Surprisingly, perhaps, similar considerations can apply in protein synthesis because it is sometimes necessary for cells to stop the production of new proteins as quickly as possible. Now, in eLife, Graham Pavitt of the University of Manchester and colleagues – including Martin Jennings as first author, Christopher Kershaw and Tomas Adomavicius – report that they have identified an 'emergency brake' for stopping protein synthesis when cells are experiencing stress (Jennings et al., 2017).

The emergency brake – which is part of the integrated stress response in cells – shuts down the process by which messenger RNA molecules are translated into primary chains of amino acids, which then fold to form active proteins. Shutting down the process that transcribes DNA to form messenger RNA would also bring protein synthesis to a halt, but not as quickly as shutting down translation can stop it. Translational control thus allows cells to respond rapidly and flexibly to external signals and various forms of stress, and failures in this process have been linked to a number of diseases: for example, mutations in an initiation protein complex called eIF2B (which is short for eukaryotic translation initiation factor 2B) cause a fatal genetic disorder of the nervous system called leukodystrophy (Pavitt and Proud, 2009; Hinnebusch, 2014; Chu et al., 2016).

The integrated stress response is an elaborate signaling pathway that stops protein synthesis in eukaryotic cells when it is activated in response to various internal and external factors (reviewed in Pakos-Zebrucka et al., 2016). The main internal factor is stress caused by the accumulation of unfolded proteins in the endoplasmic reticulum, and the external factors include viral infections and shortages of oxygen, amino acids or glucose. The integrated stress response is centered on an initiation protein complex called eIF2, and it is activated when a particular amino acid (serine 51 in its alpha subunit) is phosphorylated by a protein kinase: the identity of the kinase depends on the type of stress that the cell is responding to. This phosphorylation causes a robust reduction in general protein synthesis by blocking the initiation of translation for most messenger RNAs, but allowing the translation of selected messenger RNAs (for the production of proteins that can combat stress inside the cell).

Translation initiation begins with eIF2 binding to GTP and a molecule called initiator Met-tRNA to form a structure called the ternary complex. The role of the ternary complex is to ensure that the initiator Met-tRNA is delivered to the P-site on the ribosome and that translation begins at the correct start codon on each mRNA molecule (which involves scanning the mRNA to find the start codon, which is usually AUG). Besides eIF2, there are at least 11 other initiation factors that interact with messenger RNAs and/or subunits of the ribosome to form the so-called pre-initiation complex and ensure that translation is initiated properly. Two of these – eIF5 and eIF2B – constitute a regulatory circuit that cycles eIF2 between its active state (in which it is bound to GTP) and an inactive state (bound to GDP) that cannot initiate translation.

In some ways eIF5 resembles the accelerator of a car (Figure 1) in that it ensures that translation happens when the conditions are right for it: likewise, eIFB2 resembles a brake, slowing down the production of proteins in response to worsening conditions; and eIF2-GTP/GDP is like a clutch in that it allows the cell to change gears in response to varying conditions. However, in order to understand how these three factors perform these different roles and control protein synthesis in cells, we first need to understand the nature of their mutual interactions (for example, simultaneous versus mutually exclusive), as well as their varying affinities. It is known that eIF5 binds inactive eIF2-GDP and active eIF2-GTP with similar affinities (Algire et al., 2005), whereas initiator Met-tRNA binds eIF2-GTP with an affinity that is ~10 times greater than the affinity with which it binds eIF2-GDP (Kapp and Lorsch, 2004). Now, among other findings, Jennings et al. unexpectedly report that eIF2B binds eIF2-GDP and eIF2-GTP with similar affinities too, and that these binding affinities change in stress conditions.

**Figure 1.**
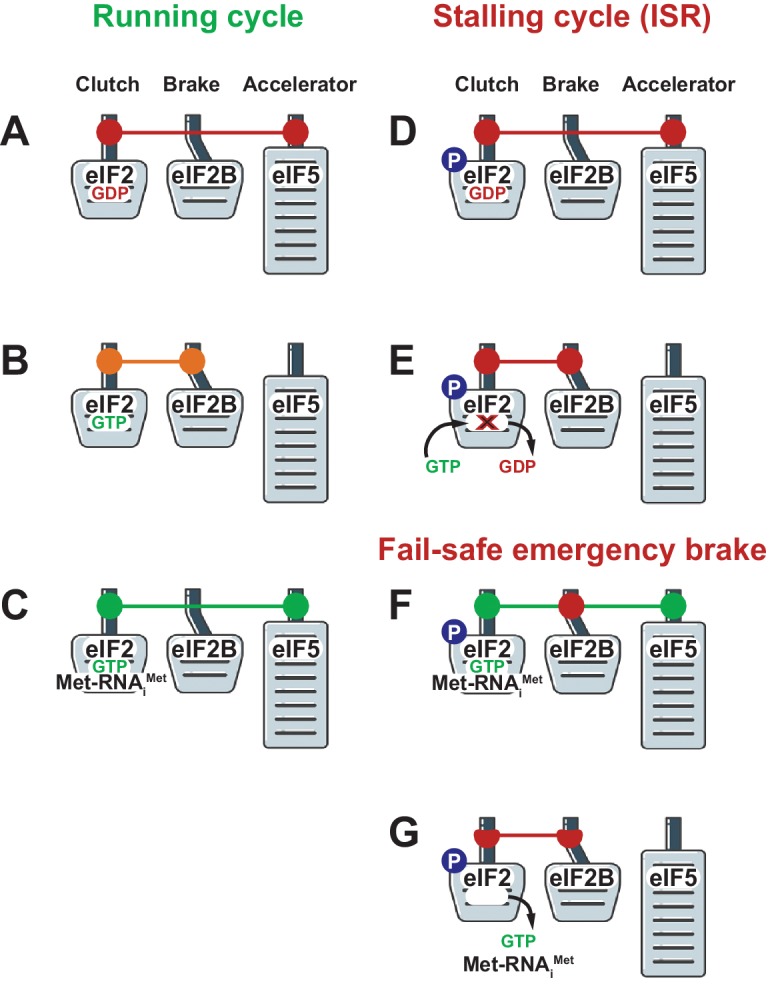
How translation can be stopped and started during protein synthesis. Three of the main players in the control of translation act like the clutch (eIF2), brake (eIF2B) and accelerator (eIF5) in a car. (**A**) Once the translation of an mRNA molecule has started, a complex containing eIF2-GDP (which is inactive) and eIF5 leaves the ribosome. (**B**) eIF2B then out-competes eIF5 and mediates the exchange of GDP and GTP to yield eIF2-GTP (which is active). (**C**) eIF2-GTP and initiator Met-tRNA then form a ternary complex, which is stabilized by eIF5, and a new cycle of translation can begin. (**D**) Sometimes a cell has to reduce protein synthesis in response to stress or other factors, and this response starts with the phosphorylation (P) of a specific amino acid (Ser51) in eIF2. (**E**) This phosphorylation has important consequences: eIF2B is unable to mediate the exchange of GDP and GTP, and translation cannot proceed. (**F, G**) Jennings et al. show that if the phosphorylation of Ser51 occurs on eIF2 present in an existing ternary complex, the phosphoryl group allows eIF2B to out-compete eIF5: this means that eIF5 cannot stabilize the ternary complex, so the complex falls apart and translation is stopped completely.

Under normal conditions, when growth is permissible, the ternary complex binds the ribosome with help of other translation initiation factors, such as eIF5 and eIF3, to start scanning for the start codon. When this codon has been found eIF5 activates GTP hydrolysis on eIF2 and the resulting eIF2-GDP molecule (which is still bound to eIF5) leaves the pre-initiation complex (Figure 1A). eIF2B then out-competes eIF5 and mediates the exchange of GDP and GTP to bring eIF2 back to its active state (Figure 1B). Initiator Met-tRNA now binds to eIF2-GTP to form a new ternary complex that – together with eIF5 – can out-compete eIF2B, and this allows a new cycle of translation to begin (Figure 1C). In other words, all three factors – eIF2, eIF2B and eIF5 – co-operate and keep the car moving.

However, when the integrated stress response is activated, the eIF2-GDP-eIF5 complex leaving the pre-initiation complex after the start codon has been recognized contains eIF2 in which serine 51 has been phosphorylated, and this results in the brakes being applied to the translation process by eIF2B. The dramatically increased strength of the binding between eIF2B and the phosphorylated eIF2-GDP means that the exchange of GDP and GTP does not occur, and that eIF5 cannot out-compete eIF2B in order to allow a new cycle of translation to begin (Figure 1E). We can think of this as the equivalent of the regular brakes on a car being used to slow it down. However, Jennings et al. show that cells have an additional 'fail-safe' emergency brake that completely stops the translation process.

The regular brake prevents the exchange of GDP and GTP on the phosphorylated eIF2-GDP-eIF5 complex as it leaves the pre-initiation complex, and thus blocks the formation of a new ternary complex: however, if the serine 51 site has been phosphorylated in the already-formed ternary complex, the emergency brake prevents it from beginning a new round of translation. It relies on eIF2B being able to out-compete eIF5 and thus destabilize the ternary complex. This way the translational vehicle is brought to a complete stop (Figure 1F, 1G).

Once we fully understand the role of eukaryotic initiation factors like eIF2, eIF2B and others in the regulation of gene expression, we will be in a better position to understand the molecular mechanisms underlying diseases caused by mutations in them.
